# Wastewater treatment from a science faculty during the COVID-19 pandemic by using ammonium-oxidising and heterotrophic bacteria

**DOI:** 10.1007/s13205-024-03961-4

**Published:** 2024-04-09

**Authors:** Lucas D. Pedroza-Camacho, Paula A. Ospina-Sánchez, Felipe A. Romero-Perdomo, Nury G. Infante-González, Diana M. Paredes-Céspedes, Balkys Quevedo-Hidalgo, Viviana Gutiérrez-Romero, Claudia M. Rivera-Hoyos, Aura M. Pedroza-Rodríguez

**Affiliations:** 1 Laboratorio de Microbiología Ambiental y Suelos, Unidad de Investigaciones Agropecuarias (UNIDIA), Grupo de Biotecnología Ambiental e Industrial (GBAI), Departamento de Microbiología, Facultad de Ciencias, Pontificia Universidad Javeriana, Carrera 7ma No 43-82, Edifício 50 Lab. 106, P.O. Box 110-23, Bogotá, DC Colombia; 2 Laboratorio de Biotecnología Aplicada, Grupo de Biotecnología Ambiental e Industrial (GBAI), Departamento de Microbiología, Facultad de Ciencias, Pontificia Universidad Javeriana, P.O. Box 110-23, Bogotá, DC Colombia; 3 Dirección de Innovación y Desarrollo, Konservo S.A.S, P.O. Box 110-23, Bogotá, DC Colombia; 4 Laboratorio de Biotecnología Molecular, Grupo de Biotecnología Ambiental e Industrial (GBAI), Departamento de Microbiología, Facultad de Ciencias, Pontificia Universidad Javeriana, P.O. Box 110-23, Bogotá, DC Colombia

**Keywords:** Ammonium-oxidising bacteria, COVID-19 outbreak, *Nitrosococcus oceani*, *Nitrosomonas europea*, *Nitrosospira multiformis*, Wastewater treatment

## Abstract

During and after the pandemic caused by the SARS-CoV-2 virus, the use of personal care products and disinfectants increased in universities worldwide. Among these, quaternary ammonium-based products stand out; these compounds and their intermediates caused substantial changes in the chemical composition of the wastewater produced by these institutions. For this reason, improvements and environmentally sustainable biological alternatives were introduced in the existing treatment systems so that these institutions could continue their research and teaching activities. For this reason, the objective of this study was to develop an improved culture medium to cultivate ammonium oxidising bacteria (AOB) to increase the biomass and use them in the treatment of wastewater produced in a faculty of sciences in Bogotá, D.C., Colombia. A Plackett Burman Experimental Design (PBED) and growth curves served for oligotrophic culture medium, and production conditions improved for the AOB. Finally, these bacteria were used with total heterotrophic bacteria (THB) for wastewater treatment in a pilot plant. Modification of base ammonium broth and culture conditions (6607 mg L^−1^ of (NH_4_)_2_SO_4_, 84 mg L^−1^ CaCO_3_, 40 mg L^−1^ MgSO_4_·7H_2_O, 40 mg L^−1^ CaCl_2_·2H_2_O and 200 mg L^−1^ KH_2_PO_4_, 10% (w/v) inoculum, no copper addition, pH 7.0 ± 0.2, 200 r.p.m., 30 days) favoured the growth of *Nitrosomonas europea*, *Nitrosococcus oceani*, and *Nitrosospira multiformis* with values of 8.23 ± 1.9, 7.56 ± 0.7 and 4.2 ± 0.4 Log_10_ CFU mL^−1^, respectively. NO_2_^−^ production was 0.396 ± 0.0264, 0.247 ± 0.013 and 0.185 ± 0.003 mg L^−1^ for *Nitrosomonas europea*, *Nitrosococcus oceani* and *Nitrosospira multiformis.* After the 5-day wastewater treatment (WW) by co-inoculating the three studied bacteria in the wastewater (with their self-microorganisms), the concentrations of AOB and THB were 5.92 and 9.3 Log_10_ CFU mL^−1^, respectively. These values were related to the oxidative decrease of Chemical Oxygen Demand (COD), (39.5 mg L^−1^), Ammonium ion (NH_4_^+^), (6.5 mg L^−1^) Nitrite (NO_2_^−^), (2.0 mg L^−1^) and Nitrate (NO_3_^−^), (1.5 mg L^−1^), respectively in the five days of treatment. It was concluded, with the improvement of a culture medium and production conditions for three AOB through biotechnological strategies at the laboratory scale, being a promising alternative to bio-augment of the biomass of the studied bacteria under controlled conditions that allow the aerobic removal of COD and nitrogen cycle intermediates present in the studied wastewater.

**Supplementary Information:**

The online version contains supplementary material available at 10.1007/s13205-024-03961-4.

## Introduction

During the COVID-19 pandemic, caused by the SARS-CoV-2 virus, the world's population had to isolate itself, most face-to-face activities stayed suspended, and biosecurity measures associated with washing, disinfection and use of personal care products were importantly increased (Ali et al. 2021; Anayah et al. 2021). This situation was assumed in all universities worldwide, determining that between the period of the first semester of both 2020 and 2021, the population of students, teachers and administrative staff had to carry out their activities remotely (Achak et al. 2021; Ministerio de Salud y Protección Social 2021; Karami et al. 2022). The 100% suspension of attendance at the different faculties resulted in less wastewater (WW), and contamination decreased. However, by the second semester of 2021 (December), the university community gradually returned to different activities resulting in increased WW volumes and changes in the chemical, physical and microbiological composition (Anayah et al. 2021). In addition, strict biosecurity measures continued to prevent virus transmission (Achak et al. 2021; Ministerio de Salud y Protección Social 2021; Wang et al. 2021) such as disinfection of teaching and research laboratories with chlorine, chlorine compounds and quaternary ammonium compounds (Achak et al. 2021; Ahmed et al. 2022). The increased use of various chemicals hurt three key aspects. In the aquatic ecosystem, the level of pollution increased and increased quantities of chlorine and ammonium-based chemicals released, which could react with each other to generate more difficult-to-remove and possibly toxic compounds, such as nitrosamines (Hora et al. 2020; Ahmed et al. 2022). In addition, chlorine could react with organic matter (humic and fulvic substances), forming trihalomethanes that have mutagenic and carcinogenic effects on aquatic biota (Hamed et al. 2017). The second negative impact was the increase in the WW of antibiotic-resistant microorganisms, which may even become a more serious public health problem caused by COVID-19, as microorganism control is not possible with a unique type of antibiotic (Ahmed et al. 2022). The third impact relates to increased amounts of disinfectants in WW when mixed with hospital wastewater. These compounds or their intermediates may affect the microorganisms performing secondary treatment (inhibition or complete removal), decreasing removal efficiency or increasing hydraulic retention times in plants (Anayah et al. 2021; Ahmed et al. 2022).

In general, a series of physical (sedimentation, filtration, degreasing, de-oiling), chemical (coagulation-flocculation, advanced oxidation), and biological (activated sludge, anaerobic digestion and biological nutrient removal) unit operations serve for wastewater treatment. These are incorporated sequentially in each treatment unit, part of the treatment plants and according to the type and final use of the wastewater. (Puri et al. 2023). The composition of WW can be classified; the micropollutants category includes analgesics, cytostatics, hormones, antibiotics, disinfectants and detergents, among others, and the macro-pollutants which are associated with chemical oxygen demand (COD), 5-day biochemical oxygen demand (BOD_5_), total suspended solids (TSS), settleable solids (SS) and some nutrients such as orthophosphates (PO_4_-P) and nitrates (NO_3_^−^), (Anayah et al. 2021). Concerning carbonaceous macro-pollutants (COD and BOD_5_), pollution is also composed of carbohydrates, proteins, oils, fats and TSS (Fatimazahra et al. 2023). For the removal of this organic matter, biological systems with aerobic, facultative or anaerobic heterotrophic microorganisms are successful (Makowska and Sowinska 2020) because they transform organic matter to produce biomass, energy and intermediates such as carbon dioxide (CO_2_) in aerobic processes and methane (CH_4_) in the anaerobic ones (Elmoutez et al. 2023; Li et al. 2023). Due to these transformations, the values of COD, BOD_5_ and TSS reduced, allowing the effluent to be discharged or reused in other domestic or agro-industrial activities (Yılmaz et al. 2020; Dinh et al. 2021; Hosney et al. 2023).

Concerning the nitrogen cycle, in the WW, the pollution is contributed by the organic matter, rich in nitrogen, proteins, urea and disinfectants derived from quaternary ammoniums (Hora et al. 2020; Achak et al. 2021; Wang et al. 2021; Ahmed et al. 2022). As a result, inorganic and organic forms are present in these wastewaters, with ammonia (NH_3_), ammonium ion (NH_4_^+^), nitrite (NO_2_^−^), nitrate (NO_3_^−^) and diatomic nitrogen (N_2_) being the most frequent inorganic forms (Han and Zhou 2022). The accumulation of nitrogen in the form of NH_4_^+^, NO_2_^−^ and NO_3_^−^, causes unpleasant odours (NH_4_^+^), and overgrowth processes of aquatic plants and the NO_2_^−^ accumulation affects water quality and can generates toxicity for aquatic animals and humans Hussein (Al-Hazmi et al. 2023).

The aerobic removal of NH_4_^+^ from wastewater occurs by nitrification and consists of two stages. The first removal stage involves the ammonium (NH_4_^+^) oxidation to hydroxylamine (NH_2_OH) by the ammonia monooxygenase (AMO, E.C. 1.14.99.39). Subsequently, hydroxylamine is oxidised to NO_2_^−^ by the hydroxylamine oxidoreductase (E.C. 1.7.2.6), (Fan et al. 2021; Zhu et al. 2022). The oxidation of NH_4_^+^ to NO_2_^−^ involves ammonium-oxidising bacteria (AOB) with genera such as *Nitrosomonas* sp*.*, *Nitrosospira* sp., *Nitrosolobus* sp., *Nitrosovibrio* sp., and *Nitrosococcus* sp., (Zorz et al. 2018; Lu et al. 2019; Aizawa et al. 2023). These bacteria are chemolithotrophic and use inorganic carbon for biomass production (Zhang et al. 2024).

NO_2_^−^ is rapidly oxidised to nitrate NO_3_^−^, by nitrite-oxidising bacteria (NOB) such as *Nitrobacter* sp., *Nitrococcus* sp. and *Nitrospira* sp., (Zorz et al. 2018; Sanders et al. 2019; Zhang et al. 2024). NO_3_^−^, can follow several transformation pathways, one of which is the assimilative reduction of NO_3_^−^ to produce NH_4_^+^; them used by microorganisms to synthesise new nitrogen-based cellular compounds, such as amino acids, peptides, proteins and nitrogenous sugars (Huang et al. 2020). Under anaerobic conditions, NO_3_^−^ is reduced by denitrification and released into the atmosphere as diatomic nitrogen (N_2_), (Huang et al. 2022; Marquez Fontalvo et al. 2022).

Recently identified alternative routes within the nitrogen cycle, such as the direct oxidation of NH_4_^+^ to NO_3_^−^ through the Comamox process, are carried out by bacteria such as *Nitrospira inopinata, Nitrospira nitrosa* and *Nitrospira nitrificans* (Ren et al. 2020a; He et al. 2022; Cui et al. 2023). These bacteria have been detected in activated sludge reactors for wastewater treatment and alternate with the autotrophic nitrification process to complement NH_4_^+^ (Ren et al. 2020b). Bacteria able to perform heterotrophic nitrification and aerobic denitrification have previously been identified in wastewater (Song et al. 2021; Zhang et al. 2023).

Despite the alternative routes discovered for the nitrogen cycle that still are under investigation (Ren et al. 2020b; He et al. 2022; Zhu et al. 2022; Gu et al. 2023), the chemolithotrophic oxidation process of NH_4_^+^ to NO_3_^−^ mediated by AOB and NOB bacteria, remains the most reported route for aerobic ammonium removal in wastewater and regulates the accumulation and transformation of NH_4_^+^ (He et al. 2022). Because AOB bacteria are in varying concentrations in WW and grow more slowly than other microorganisms that are part of the microbial communities in an activated sludge reactor (Xia et al. 2018; Yang et al. 2020; Zhang et al. 2024), ammonium oxidation proceeds more slowly than oxidation of carbonaceous organic matter, causing hydraulic retention times prolonged (Soliman and Eldyasti 2018; Yin et al. 2018; Cui et al. 2023).

Different biotechnological strategies allow for an increase in AOB populations, such as stimulating the populations present in WW, regulating the initial NH_4_^+^ concentration and or adding carbonate-based salts to provide an alternative source of CO_2_ for AOB (Campbell et al. 2011; Bennett et al. 2016; Cui et al. 2023; Zhang et al. 2024). On the other hand, the biological reactor operating setup could be improved by regulating pH and increasing the aeration rate (Soliman and Eldyasti 2018; Yin et al. 2018; Cui et al. 2023; Zhang et al. 2024).

A less explored alternative is to isolate AOB from natural ecosystems or to acquire AOB from reference collections, grow them under controlled conditions inoculate it to WW. Co-inoculation of more than one AOB at high concentrations ensures a shorter adaptation time of the bacteria to WW, ammonium oxidation from the beginning of the process and survival of the same AOB for extensive times (Zhou et al. 2020; Moloantoa et al. 2022). Also, AOBs develop cooperative interactions with WW heterotrophic and photosynthetic microorganisms to receive mutual benefit and improve NH_4_^+^ removal efficiency (Zhou et al. 2020; Sun et al. 2021). In this interaction, the heterotrophic microorganisms produce the CO_2_ necessary for the AOBs to obtain their inorganic carbon source, and after oxidising NH_4_^+^, they decrease the toxic effect of this compound for the heterotrophic microorganisms (Zhou et al. 2020; Fan et al. 2021). Finally, WW microorganisms may also include bacteria that oxidise NO_2_^−^ to NO_3_^−^; the NO_3_^−^ is reduced by the microorganisms from the WW by assimilative pathway or can be recovered through different physical or chemical processes as a strategy for recycling nutrients from wastewater (Cruz et al. 2018; Spasov et al. 2020; Li et al. 2021; Sun et al. 2021; Moloantoa et al. 2022).

The objective of this research was to improve a culture medium and the selection of operating conditions at the laboratory scale to increase the biomass production of three ammonium-oxidising bacteria (AOB) (*Nitrosomonas europea*, *Nitrosococcus oceani* and *Nitrosospira multiformis*). Bacteria assayed for the NH_4_^+^ oxidation and organic matter removal in cooperation with heterotrophic microorganisms from the WW generated at the Faculty of Sciences in Bogotá, D.C., Colombia, also with the intention that other universities in the world can implement and improve the same or similar removal system for wastewater produced during teaching and research activities.

## Materials and methods

### Preservation of bacteria and growth conditions

Three genera of ammonium-oxidising bacteria (AOB) purchased from the American Type Culture Collection, *Nitrosomonas europea* ATCC 25978™ (https://www.atcc.org/products/25978, 2022), *Nitrosococcus oceani* ATCC 19707™ (https://www.atcc.org/products/19707, 2022) and *Nitrosospira multiformis* ATCC 25196™ (https://www.atcc.org/products/25196, 2022) were used in this study. The three bacteria were preserved in ammonium broth, supplemented with 30% (w/v) glycerol and frozen at − 20 ± 2 °C, following the methodology previously described (Amador et al. 1994; Poutou et al. 1994). To Master Cell Banks (MCBs) stability analysis, periodic sampling (in triplicate) was for five months performed; at each sampling interval, vials of each bacterium were thawed and tr. ansferred to 100 mL Erlenmeyer flasks containing 20 mL of ammonium sulphate broth base (500 mg L^−1^ (NH_4_)_2_SO_4_, 40 mg L^−1^ MgSO_4_·7H_2_O, 40 mg L^−1^ CaCl_2_·2H_2_O, 200 mg L^−1^ KH_2_PO_4_, 2.0 mg L^− 1^ CaCO_3_, pH 7.0 ± 0.2). The Erlenmeyer flasks incubation conditions were 15 days at 26 ± 2 °C, 120 ± 2 r.p.m.; the response variables analysed were morphological purity (Gram staining), cell viability (surface seeding on ammonium agar) (Martínez-Salgado et al. 2013) and NH_4_^+^ to NO_2_^−^ oxidation using HACH methods number 8155 and 8153, respectively (Hach Company/Hach Lange GmbH 2020a, 2022).

### Plackett Burman experimental design (PBED)

To modify the component concentration of the ammonium sulphate base broth, reported by Martínez-Salgado et al., (2013) and the culture conditions to favour biomass production and oxidation of NH_4_^+^ to NO_2_^−^ (mg L^−1^) the *Nitromonas europea* strain was used. The study involved a PBED, which generated 12 treatments in triplicate for the factors and levels evaluated (Table 1). For each treatment preparation, ammonium sulphate broth was formulated according to the combinations of each treatment, using 100 mL Erlenmeyers, each one with 20 mL of broth (Effective Working Volume, EWV 1/5); Erlenmeyers inoculation was with a suspension of *N. europea* previously grown in base ammonium broth for 15 days at 26 ± 2 °C, 120 ± 2 r.p.m. The cells were washed three times with 0.85% (w/v) saline and centrifuged at 5000 r.p.m., for 10 min to remove traces of NH_4_^+^, NO_2_^−^ and NO_3_^−^. The volumetric percentage of the inoculum in each Erlenmeyer was performed according to the experimental design, maintaining an initial concentration of 1.0 × 10^3^ ± 1.0 × 10^1^ CFU mL^−1^ (Colony Forming Unit mL^−1^). Each treatment settings, such as culture time (days) and agitation speed (r.p.m.), were according to the different combinations adjusted (Table 1). The response variables were, colony count on ammonium base agar, expressed as Log_10_ CFU mL^−1^, (Martínez-Salgado et al. 2013) and NO_2_^−^ concentration determined by the Griess-Illosvay method at *λ*_520 nm_ using distilled water and 100 μL of Griess reagent as blank (Schmidt and Belser 1994). Design Expert V7.0 and SAS V9.0 for Windows allowed the analysis of the results with a confidence level of 95% following a methodology previously reported (Blanco-Vargas et al. 2021).

**Table 1:** Plackett–Burman experimental design (PBED) to determine the best culture media and operating conditions for ammonia-oxidizing bacteria *Nitrosomonas europea* ATCC 25978 (AOB)

Treatments	Factor A	Factor B	Factor C	Factor D	Factor E	Factor F	Factor G
	(NH_4_)_2_SO_4_ (mg L^−1^)*	pH	CuSO_4_ (mg L^−1^)	Time (day)	Inoculum (% v/v)	CaCO_3_ (mg L^−1^)	Agitation (r.p.m.)
1	6607	5.0	3.19	15	1	84.1	120
2	6607	7.0	0.00	30	1	0.0	120
3	2643	7.0	3.19	15	10	0.0	120
4	6607	5.0	3.19	30	1	0.0	200
5	6607	7.0	0.00	30	10	84.1	120
6	6607	7.0	3.19	15	10	0.0	200
7	2643	7.0	3.19	30	1	84.1	200
8	2643	5.0	3.19	30	10	84.1	120
9	2643	5.0	0.00	30	10	0.0	200
10	6607	5.0	0.00	15	10	84.1	200
11	2643	7.0	0.00	15	1	84.1	200
12	2643	5.0	0.00	15	1	0.0	120

*6607 mg L^−1^ de (NH_4_)_2_SO_4_ (1800 mg L^−1^ NH_4_-N). 2643 mg L^−1^ de (NH_4_)_2_SO_4_ (720,2 mg L^−1^ NH_4_-N). Each of the treatments was supplemented with 40 mg L^−1^ MgSO_4_·7H_2_O, 40 mg L^−1^ CaCl_2_·2H_2_O and 200 mg L^−1^ KH_2_PO_4_

### Growth curves of *Nitrosomonas europea*, *Nitrospira multiformis* and *Nitrosococcus oceani* in modified ammonium sulphate broth

For the performance of the three AOB growth curves, we used the selected treatment in the PBED. Curves were performed in triplicate in 2.0 L Erlenmeyer with 0.6 L EWV of modified ammonium sulphate broth (T5 of PBED). Curves inoculation was 10% (v/v) suspension of each bacterium with an initial concentration of 1.0 × 10^3^ ± 1.0 × 10^1^ CFU mL^−1^. Culture conditions were temperature 30 ± 2 °C, agitation 200 ± 5 r.p.m, initial pH 7.0 ± 0.2 and 30 days of evaluation. Periodic sampling allows for determining residual NH_4_^+^ concentration (mg L^−1^), (McCrady 1996), nitrifying bacteria count on modified ammonium sulphate agar (Log_10_ CFU mL^−1^), (Martínez-Salgado et al. 2013), pH and NO_2_^−^ concentration (mg L^−1^), (Schmidt and Belser 1994). For the calculation of the kinetic parameters such as biomass yield from ammonium (*Y*_*X*/NH4_), nitrite yield from ammonium (*Y*_NO2/NH4_), biomass productivity (*P*_*x*_) and nitrite productivity (P NO_2_) used the following previously reported Eqs. (1–4) (Doran 2013; Blanco-Vargas et al. 2021).

Biomass yield from ammonium: (*Y*_*X*/NH4_).

1 $$ Y_{(X/S)} = \frac{{{\text{CFU}}_f - {\text{CFU}}_i }}{S_0 - S_f } $$

where *Y*_(*x*/*s*)_: is the biomass yield from substrate (Ammonium) (CFU mL^−1^ mg^−1^), CFU_*f*_: is the final colony forming units (CFU mL^−1^), CFU_*i*_: is the initial colony forming units (CFU mL^−1^), *S*_o_: is the initial substrate concentration (mg mL^−1^), *S*_*f*_: is the final substrate concentration (mg mL^−1^).

Yield of NO_2_/ NH_4_.^+^: (*Y*_NO2/NH4_)

2 $$ Y_{(P/S)} = \frac{P_f - P_i }{{S_0 - S_f }} $$

where *Y*_(*P*/*S*)_: is the yield of product (nitrite) from substrate (ammonium) (mg mg^−1^), *P*_*f*_: is the final product concentration (mg L^−1^), *P*_*i*_: is the initial product concentration (mg L^−1^), *S*_0_: is the initial concentration of substrate (mg L^−1^), *S*_*f*_: is the final concentration of substrate (mg L^−1^).

Biomass productivity *P*_(*X*)_

3 $$ {\text{Biomass}}\;{\text{Productivity}} = \frac{X_f - X_0 }{T} $$

where *X*_*f*_: is the final biomass concentration (CFU mL^−1^), *X*_0_: is the initial biomass concentration (CFU mL^−1^), *T*: is the time when the maximum biomass production has been reached.

Nitrite productivity (P_NO2_)

4 $$ {\text{Nitrite}}\;{\text{Productivity}} = \frac{P_f - P_0 }{T} $$

where *P*_*f*_: is the final product concentration (mg L^−1^), *P*_0_: is the initial product concentration (mg L^−1^), *T* is the time when the highest amount of product has been reached.

#### NH_4_^+^ removal curves in wastewater (WW)

##### Sampling and characterisation of WW

To evaluate the three bacteria (consortium) as an alternative for NH_4_^+^ removal, wastewater (WW) generated by a biological sciences faculty at Bogotá, D.C., Colombia served. Sampling moments were during six different times, the first in December 2019 (before pandemic isolation), the second in July 2020 (under total isolation because of the COVID-19 pandemic), the third in December 2020 (under isolation because of the pandemic), the fourth in April 2021 (under total isolation by pandemic), the fifth in August 2021 (under partial return to presential) and the sixth in December 2021 (after total return to presential activities). Samples were taken from an inspection box, located in one of the busiest areas of the faculty and subjected to physical, chemical and microbiological analyses (Table 2).

**Table 2:** Physical, chemical and microbiological analysis of wastewater

Parameter	Units	Tecniques	References
pH	Und	Electrode determination	Baird and Bridgewater (2017)
SS	mL L^−1^	Sedimentation	
SST	mg L^−1^	Gravimetry	
COD	mg L^−1^	Closed reflux and digestion with dichromate and sulphuric acid	Hach Company/Hach Lange GmbH (2020b)
BOD_5_	mg L^−1^	Incubation for 5 days and membrane electrode	Baird and Bridgewater (2017)
NH_4_^+^	mg L^−1^	Salicylate method	Hach Company/Hach Lange GmbH (2022)
NO_2_^−^	mg L^−1^	Ferrous sulphate method	Hach Company/Hach Lange GmbH (2020a)
NO_3_^−^	mg L^−1^	Cadmium reduction method	Hach Company/Hach Lange GmbH (2014)
Thermotolerants Coliforms	mg L^−1^	Most likely number MLN/100 mL	Vesga et al. (2019)
Total coliforms TC	CFU mL^−1^	Chromogenic agar count	Rojas-Higuera et al. (2010)
*Escherichia coli*	CFU mL^−1^	Chromogenic agar count	
Total heterotrophic bacteria THB	mg L^−1^	Nutrient agar count	Martínez-Salgado et al. (2013)
Ammonium-oxidising bacteria AOB	mg L^−1^	Ammonium agar count	

### Setup and evaluation of the biological reactor coupled with secondary sedimentation

The WW used for the removal experiments in the biological reactor corresponded to the sampling of the second half of 2021. Two types of removal curves in triplicate were used; the first included the addition of a consortium of AOB (*N. europea*, *N. oceiani* and *N. multiformis*), previously produced in the modified ammonium sulphate broth (T5 of the PBED), and also the microorganisms from the WW (heterotrophic bacteria, total coliforms, *Escherichia coli* and AOB bacteria); this curve acronym was AOB and WW bacteria. Likewise, three other removal curves were carried out that did not include the AOB bacteria consortium and only evaluated the removal capacity of the WW microorganisms; this curve acronym was WW bacteria. All curves used the same biological reactor setup.

A homogenisation tank with 10 L of non-sterile WW allows for pH adjustment (7.0 ± 0.3), temperature measurement (19 ± 2 °C) and aeration for 10 min. It was then transferred with a submersible pump to the grease trap to reduce the possible presence of fats and oils. By gravity, the WW passed to the biological reactor, which contained 2.0 L of the consortium of the three AOB (1:1:1 ratio) previously produced for 15 days at 30 ± 2 °C and 200 ± 5 r.p.m. in modified ammonium sulphate broth and whose initial concentration was 1.0 × 10^3^ ± 1.0 × 10^1^ CFU mL^−1^. The air (non-sterile) supply was with air pumps at a rate of 1.0 L min^−1^. Once the WW was homogenised and aerated for 10 min, initial sampling was performed every 24 h until 96 h. At the end of the hydraulic retention time in the biological reactor, the effluent passed to the secondary settling tank for 1 h and then recovered to determine the output parameters at 97 h (Pedroza-Camacho et al. 2018; Puentes-Morales et al. 2021). For the curves with only WW bacteria, 2.0 L of WW replaced the 2.0 L of the consortium to complete the TEV of the biological reactor.

## Results

### Preservation of bacteria and growth conditions

The preservation in ammonium broth supplemented with 30% (w/v) glycerol at − 20 °C guaranteed the purity, viability and activity of the three bacteria in all experiments. *N. europea* ATCC 25978™ grew on ammonium agar forming small, punctate colonies observed with a slight blue hue in the light. Gram staining showed Gram-negative cocci (Soliman and Eldyasti 2018); (https://www.atcc.org/products/25978, 2022), (Fig. S1a and b Supplementary Material). *N. oceani* ATCC 19707™ formed small, punctate, translucent colonies. Gram staining showed Gram-negative cocci (https://www.atcc.org/products/25978, 2022), (Fig. S2a and b Supplementary Material). *N. multiformis* ATCC 25196™ grew to form small, punctate, translucent colonies, and Gram-negative short spiral bacilli were observed (https://www.atcc.org/products/25978, 2022), (Fig. S3a and b Supplementary Material).

The initial concentration of the inoculum for MCBs was 5.8 ± 0.0, 5.7 ± 0.0 and 5.7 ± 0.0 Log_10_ CFU mL^−1^, for *N. europea, N. oceani* and *N. multiformis*, respectively. In these inoculums, the residual NH_4_^+^ concentration was 275 ± 3, 272 ± 5 and 381 ± 4 mg L^−1^ for *N. europea, N. oceani* and *N. multiformis*. NO_2_^−^ production was 0.272 ± 0.03, 0.211 ± 0.09 and 0.106 ± 0.078 mg L^−1^, for *N. europea, N. oceani* and *N. multiformis*, respectively. These results showed that the three bacteria were at high concentrations and that ammonium oxidation was satisfactory to produce the inoculum for 15 days to perform bacterial preservation.

**Fig. 1:**
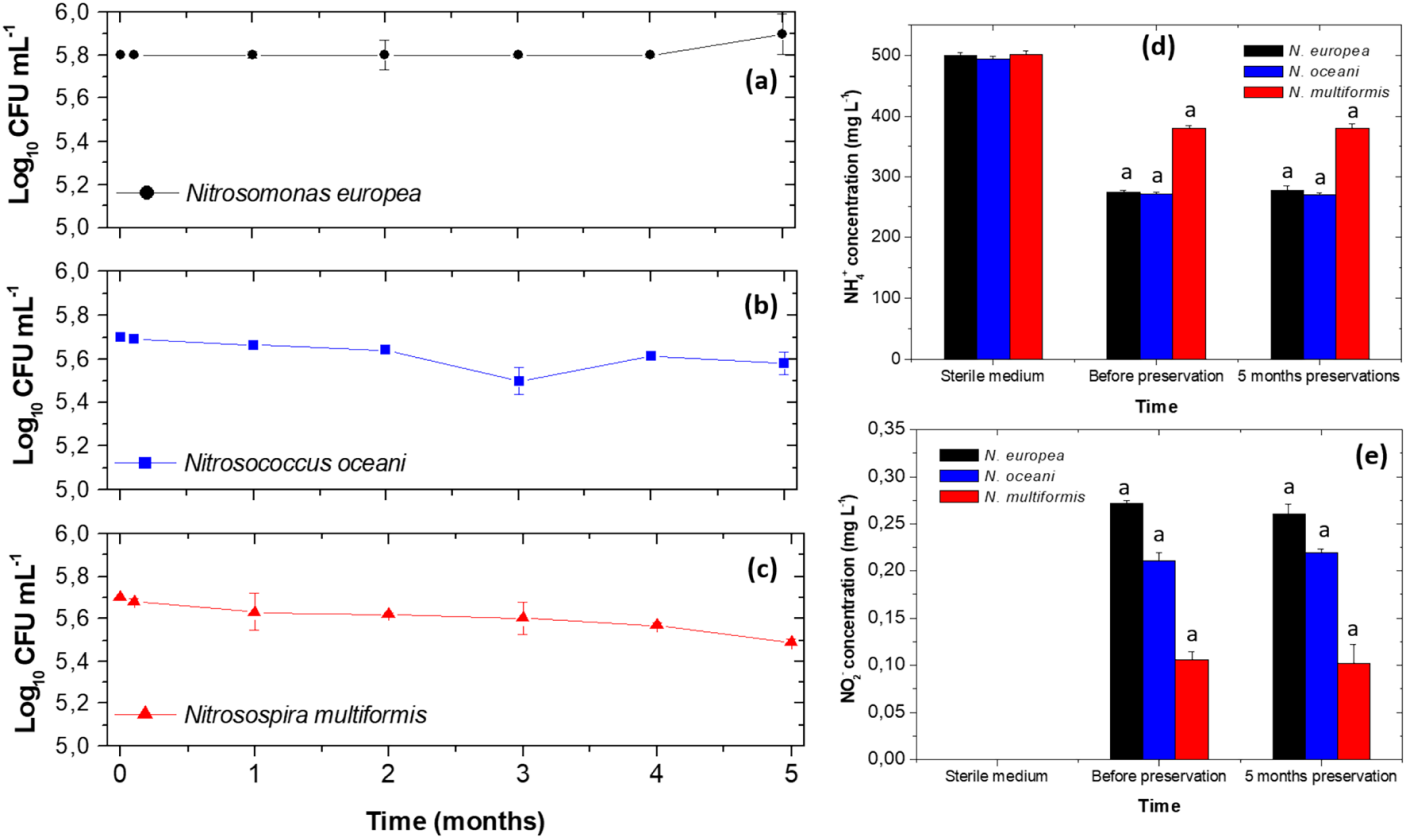
**a** Viability assessment for *N. europea* **b** Viability assessment for *N. oceani* **c** Viability assessment for *N. multiformis* **d** NH_4_^+^ concentration **e** NO_2_^−^ concentration for MCB preserved in ammonium broth supplemented with 30% (w/v) glycerol, − 20 ± 2.0 °C and 5 months. Average of three replicates with respective standard deviation

Once occurred the culture in ammonium broth rapidly mixture with 87% (w/v) glycerol to a final concentration of 30% (w/v) glycerol, no decrease in CFU was observed, even after 24 h of preservation (Fig. 1a, b and c). In the subsequent sampling count decrease occurred, but it was lower than a Log unit, and at the end of five months of preservation, counts were 5.6 ± 0.23, 5.58 ± 0,65 and 5.5 ± 0.1 Log_10_ CFU mL^−1^, for *N. europea, N. oceani* and *N. multiformis*, respectively (Fig. 1a, b and c).

On the other hand, the ability to oxidise NH_4_^+^ to NO_2_^−^ lasted throughout the five preservation months, since when cultured in ammonium sulphate broth (initial concentration: 500 ± 5 mg L^−1^) for 15 days, the three strains oxidised the substrate reaching final concentrations of 279 ± 6, 270 ± 3 and 380 ± 7 mg L^−1^ for *N. europea*, *N. oceani* and *N. multiformis* (Fig. 1d).

NO_2_^−^ concentrations were 0.261 ± 0.010 mg L^−1^, 0.222 ± 0.030 mg L^−1^ and 0.102 ± 0.020 mg L^−1^ for *N. europea*, *N. oceani* and *N. multiformis,* respectively (Fig. 1e). Once compared the results with NH_4_^+^ and NO_2_^−^ concentrations of the three bacteria before preservation, and no significant differences (*p* > 0.0001) occurred before and after freezing of the ammonium broth with 30% (w/v) glycerol at − 20 °C (Fig. 1e, d).

### Plackett Burman experimental design (PBED)

According to the analysis of variance, the model for AOB count was significant (*p* = 0.0472), allowing us to evaluate the effect of the factors on this response variable. The correlation coefficient *R*^2^ was 0.8981, and the Adq. Precision was 5.83 (value > 4.0). The lack of fit value was not significant (*p* = 0.234), indicating that the model space is navigable (Table 3).

**Table 3:** Plackett–Burman experimental design (PBED), to improve the culture medium and operating conditions for NO_2_^−^ production and colony count using *Nitrosomonas europea*

Factor	NO_2_^−^ concentration (mg L^−1^)						Colony counts (CFU mL^−1^)					
	CS^a^	FD^b^	MS^c^	F value	Prob > f	RC^d^	CS^a^	FD^b^	MS^c^	F value	Prob > f	RC^d^
Model	512.93	7	73.28	4.86	**0.0472**	10.01	7.17	7	1.02	4.17	0.0929	− 1.69
A	81.11	1	81.11	5.38	**0.0491**	**2.60**		1	0.51	2.06	0.2246	− 0.21
B	263.26	1	263.26	17.46	**0.0139**	**4.68**	0.51	1	4.55	18.53	**0.0126**	**0.62**
C	86.48	1	86.48	5.74	0.0747	2.68	4.55	1	0.74	3.00	0.1581	− 0.25
D	7.03	1	7.03	0.47	0.5323	− 0.77	0.74	1	0.053	0.22	0.6655	0.067
E	32.69	1	32.69	2.17	0.2149	1.65	0.053	1	0.41	1.69	0.2638	− 0.19
F	42.22	1	42.22	2.80	0.1695	1.88	0.41	1	0.85	3.47	0.1358	− 0.27
G	0.14	1	0.14	0.0095	0.9269							
	0.11	0.85	1	0.055	0.22		0.067					
Adj R-Squared	0.8981						0.7782					
Adequate precision	5.832						5.871					
Lack of Fit	0.234						0.134					

Bold values of Prob > f less than 0.0500 indicate model terms are significant

^a^*CS* Cuadratic sum

^b^*FD* Freedon degress

^c^*MS* Media square

^d^*RC* Regression coefficient

The influential factors for NO_2_^−^ production was A and B (ammonium sulphate concentration and pH), with *p*-values of 0.0491 and 0.0139, respectively. For factors A and B, the regression coefficients were 2.60 and 4.68, indicating that one should work with the high levels of each factor (6607 mg L^−1^ of (NH_4_)_2_SO_4_ and pH 7.0), (Table 3).

For AOB colony count variable, the model was not significant (*p* = 0.0929), the correlation coefficient *R*^2^ was 0.7782 and the Adq. precision was 5.5.8 (value > 4.0). The only significant factor was pH (Factor B) *p* = 0.0126 and a regression coefficient of 0.62. This value suggests that the increase in colony count was favourable at pH 7.0 (Table 3).

**Fig. 2:**
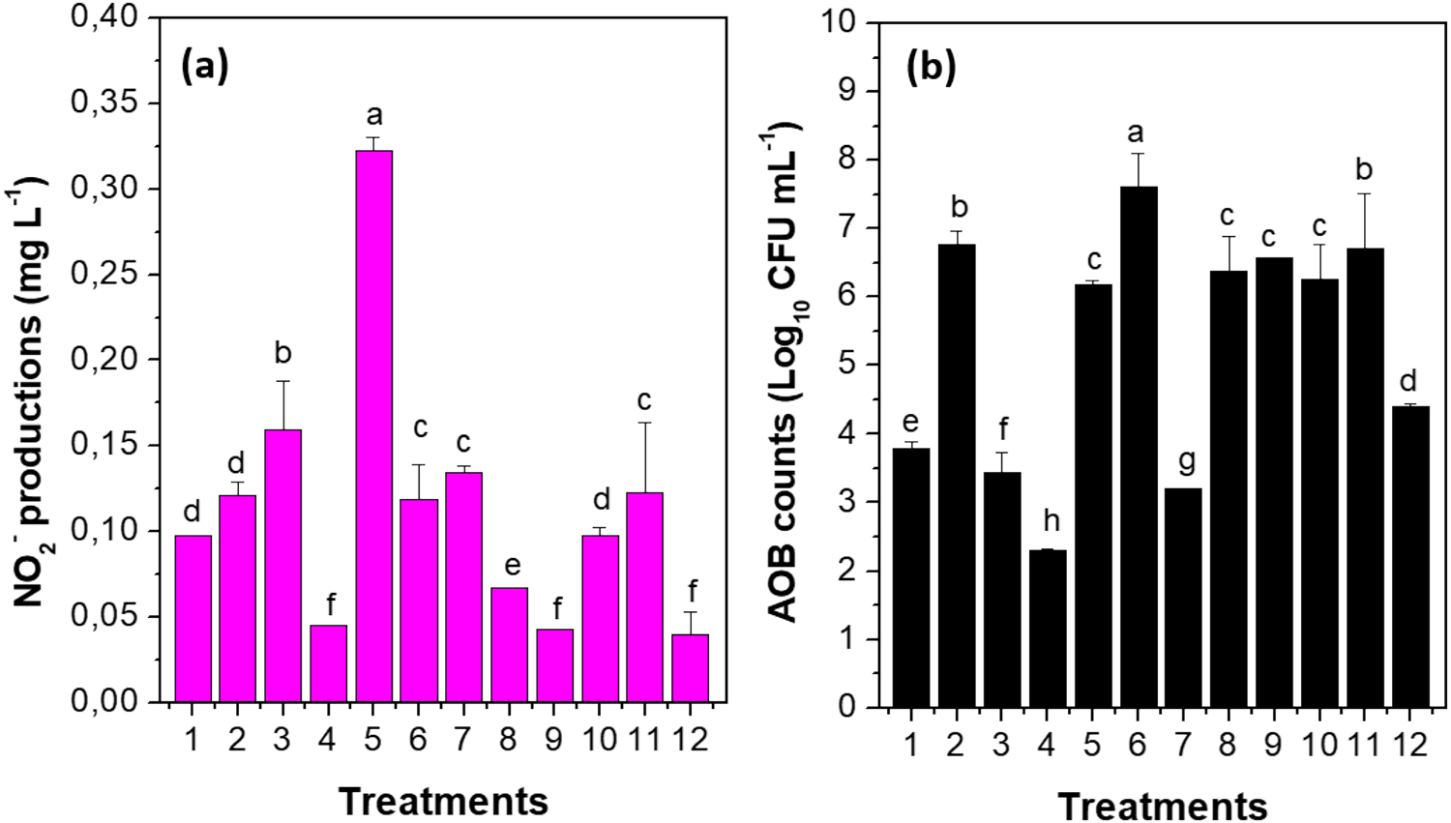
Mean comparison among treatments from PBED: **a** NO_2_^−^ production; **b** AOB bacteria count presented as Log_10_ CFU mL^−1^; letters represent heterogeneous groups according to statistical analyses (same letter for more than one experiment means no statistical differences); A corresponds to the treatments with the best NO_2_^−^ production and AOB bacteria counts in order by B, C, D, E and F

Once calculated that factors A and B influence the response variables, a means comparison between the 12 treatments resulted in significant differences (*p* < 0.0001). Regarding NO_2_^−^ concentration, the highest concentration occurred in T5, with values of 0.323 ± 0.003 mg L^−1^, followed by T3 and T11 (0.121 ± 0.038 and 0.122 ± 0.050 mg L^−1^), (Fig. 2a).

Concerning the colony count (Log_10_ CFU mL^−1^), significant differences resulted between treatments (*p* < 0.0001). The highest was the treatment T6 with 7.0 Log_10_ CFU mL^−1^; however, in this treatment, the NO_2_^−^ production was not higher than 0.2 mg L^−1^; the reason for which, it does not continue in the study. On the contrary, T5, in which the NO_2_^−^ concentration was the highest, and the biomass count was 6.2 Log_10_ CFU mL^−1^ continues in the study. Although the count value was the highest in T6, the difference with T5 was 1.4 Log_10_ CFU mL^−1^ (Fig. 2b). Therefore, the selected conditions for the growth curves were 6607 mg L^−1^ of (NH_4_)_2_SO_4_, 84 mg L^−1^ CaCO_3_, 10% (w/v) inoculum, no copper addition, pH 7.0 ± 0.2, 200 r.p.m., 30 days. Additionally, 40 mg L^−1^ MgSO_4_·7H_2_O, 40 mg L^−1^ CaCl_2_·2H_2_O and 200 mg L^−1^ KH_2_PO_4_ were added to the medium.

### Growth curves in modified ammonium broth using *Nitrosomonas europea*, *Nitrospira multiformis* and *Nitrosococcus oceani*

**Fig. 3:**
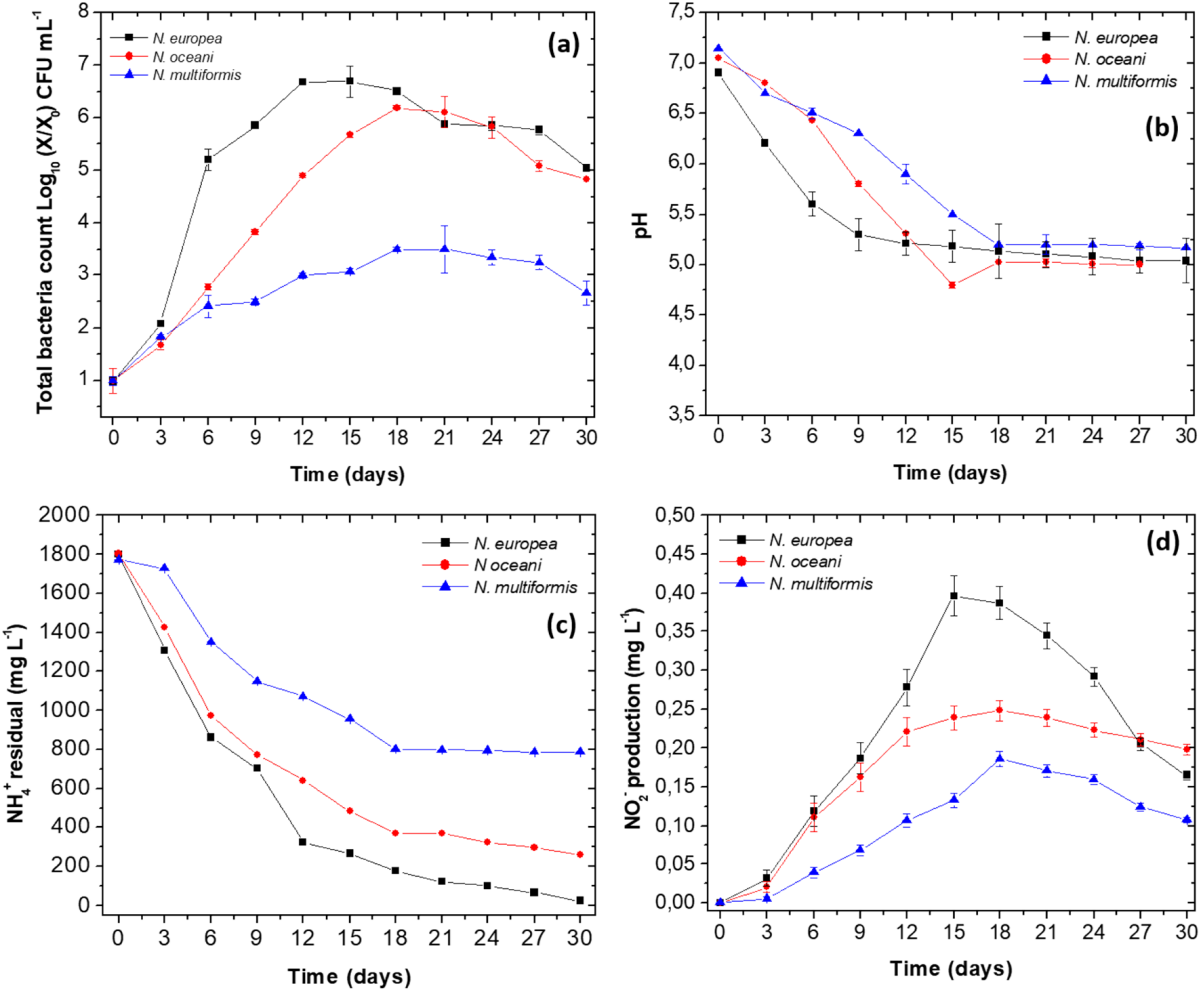
Growth curve for *N. europea*, *N. oceani* and *N. multiformis* for 30 days at 30 °C and 200 r.p.m. **a** Colony count in Log_10_ X/X_0_ CFU mL^−1^). **b** pH. **c** Residual NH_4_^+^ in mg L^−1^. **d** NO_2_^−^ production in mg L^−1^. Results are the average of three replicates with their respective standard deviation

Growth curves for each bacterium follow the formulation and conditions selected at T5 of the PBED design. The adaptation phase was not observed in any of the three bacteria between time zero and the first three days (Fig. 3a). After the third culture day, growth tendency varies for each bacterium; the exponential growth for *N. europea* prolonged until 15 days after obtaining 8.23 ± 1.9 Log_10_ CFU mL^−1^ (Fig. 3a). On the other hand, the exponential phase of *N. oceani* lasted until 18 days, with of 7.56 ± 0.7 Log_10_ UFC mL^−1^ (Fig. 3a). The bacterium that grew more slowly was *N. multiformis*, its exponential phase lasted until 21 days, and the count obtained was 4.2 ± 0.4 Log_10_ UFC mL^−1^ (Fig. 3a). Concerning pH changes, a decrease in pH value (initial 7.0 ± 0.2) was observed in all three growth curves, ending in values of 5.04 ± 0.22, 4.8 ± 0.03 and 5.17 ± 0.78 for *N. europea*, *N oceani* and *N. multiformis* (Fig. 3b).

The initial NH_4_^+^ concentrations were 1799, 1802 and 1772 mg L^−1^, for *N. europea*, *N oceani* and *N. multiformis*, respectively. The first two bacteria consumed more NH_4_^+^ and in a shorter time to finish at 26 and 260 mg L^−1^ after 30 days. *N. multiformis* consumed NH_4_^+^ lowly, and the residual concentration was 785 mg L^−1^ (Fig. 3c). As a product of NH_4_^+^ oxidation, NO_2_^−^ was produced with *N. europea* being the most efficient, as the 15-day production was 0.396 ± 0.0264 mg L^−1^. *N. oceani* produced a maximum NO_2_^−^ of 0.247 ± 0.013 mg L^−1^ at 18 days, and *N. multiformis* only produced 0.185 ± 0.003 mg L^−1^ at 19 days (Fig. 3d).

The optimal production time for each of the bacteria, the yields and the productivities are in Table 4. The biomass/substrate yield indicated that *N. europea* was the bacterium that produced the most biomass at the expense of NH_4_^+^ (3.1 × 10^7^ ± 2.1 × 10^2^ CFU mL^−1^ mg^−1^ L^−1^); also, the biomass and NO_2_^−^ productivity was the highest with values of 1.1 × 10^7^ ± 2.4 × 10^1^ CFU mL^−1^ d^.−1^ and 0.0264 ± 0.012 mg L^−1^ d^−1^. Hight biomass and NO_2_^−^ yield (6.8 × 10^6^ ± 3.5 × 10^1^ CFU mL^−1^ mg^−1^ L^−1^ and 2.5 × 10^–4^ ± 1.5 × 10^–2^ mg mg^−1^) and productivities (*P*_*x*_ 1.1 × 10^7^ ± 2.4 × 10^1^ CFU mL^−1^ d^−1^ and *P*_NO2_ 0.0264 ± 0.012 mg L^−1^ d^−1^) were also obtained with the *N. oceani* strain but at 18 days of culture. For *N. multiformis*, the highest yields and productivities appeared up to 21 days (Table 4).

**Table 4:** Kinetic production parameters for each strain in modified ammonium broth

Parameter	*N. europea* 15 d	*N. oceani* 18 d	*N. multiformis* 21 d
Y X/NH_4_ (CFU mL^−1^ mg^−1^ L^−1^)	3.1 × 10^7^ ± 2.1 × 10^2a^	6.8 × 10^6^ ± 3.5 × 10^1b^	4.7 × 10^3^ ± 2.1 × 10^1c^
Y NO_2_/NH_4_ (mg mg^−1^)	2.5 × 10^–4^ ± 1.5 × 10^−2a,b^	1.7 × 10^–4^ ± 2.0 × 10^−2a,b^	1.9 × 10^–3^ ± 3.0 × 10^−1c^
*P*_*x*_ (CFU mL^−1^ d^−1^)	1.1 × 10^7^ ± 2.4 × 10^1a^	2.0 × 10^6^ ± 2.5 × 10^2b^	7.2 × 10^2^ ± 3.1 × 10^1c^
*P*NO_2_ (mg L^−1^ d^−1^)	0.0264 ± 0.012^a^	0.0137 ± 0.023	0.0081 ± 0.0023

Lowercase letters within rows and columns represent significant differences between strains associated with kinetic parameters

#### NH_4_^+^ removal curves in wastewater

##### Sampling and characterisation of WW

Table 5, shows the results of the analyses performed on the six WW batches; in the December 2019 sampling (before confinement), the parameters were in low concentrations, especially for COD, BOD_5_, NH_4_^+^, NO_2_^−^, NO_3_^−^ and microorganism counts. During July and December 2020, the effect of COVID-19 containment on most of the parameter concentrations generates the lowest values of all samplings. In April and June 2021, a slight increase in all parameters occurred, corresponding to progress in COVID-19 vaccination and some degree of decrease in hospitalisation, leading to a partial return to the presence and higher numbers of people in circulation (Table 5). The highest COD, BOD_5_, SST, SS, NH_4_^+^, NO_2_^−^, NO_3_^−^ and microorganism counts appeared in the last sampling from December 2021, a time when all professors, administrative and part of the student staff had returned to the faculty from 8:00 am to 5:00 pm (Table 5).

**Table 5:** Physical, chemical and microbiological analyses performed on domestic wastewater during isolation by COVID-19

Parmeter	Units	M-1	M-2	M-3	M-4	M-5	M-6	Resolution 0631*	Resolution 1207**	EPA*** Title-40/chapter-I/subchapter-N
pH	Und	8.16 ± 0.87	7.83 ± 1.1	8.1 ± 0.9	6.7 ± 0.2	8.8 ± 0.5	7.88 ± 0.9	6.00 a 9.00	6.00 a 9.00	6.0–9.0
SS	mL L^−1^	3 ± 0	1.0 ± 1	1.0 ± 1	1.0 ± 1	1.0 ± 1	4.0 ± 0.7	1.50	Analysis and reporting	1
SST	mg L^−1^	12 ± 1.04	4.2 ± 0.7	2.2 ± 0.5	12 ± 1	7.45 ± 1.23	325 ± 12	75		45
COD	mg L^−1^	135 ± 4	18 ± 2	25 ± 2	255 ± 6	217 ± 6	697 ± 27	225		NE
BOD_5_	mg L^−1^	106 ± 6	11.0	14 ± 3	101 ± 6	69 ± 3	325	75	30	45
COD/BOD_5_ ratio		0.780	0.61	0.56	0.4	0.31	0.46	NE	NE	NE
NH_4_^+^	mg L^−1^	0.9 ± 0.04	1.2 ± 0.9	3.6 ± 0.8	10 ± 0.06	20 ± 0.06	43 ± 6	Analysis and reporting	Analysis and reporting	7
NO_2_^−^	mg L^−1^	< 0,0004	< 0.007	1.7 ± 0.2	2.3 ± 0.092	1.2 ± 0.04	3.0			NE
NO_3_^−^	mg L^−1^	0.02 ± 0.006	0.1	0.9 ± 0.1	1.6 ± 0.03	0.4 ± 0.05	8.5		5.0	
Thermotolerants Coliforms TC	CFU mL^−1^	4.0 × 10^2^ ± 1.0 × 10^1^	3.0 × 10^2^ ± 1.0 × 10^1^	1.0 × 10^3^ ± 1.0 × 10^1^	12 × 10^5^ ± 1 × 10^1^	2.6 × 10^3^ ± 1 × 10^1^	4.1 × 10^4^ ± 1 × 10^1^	Report for mass loadings greater than 125 kg d^−1^ of BOD_5_	1.0 × 10^4^	ND
*E. coli*	CFU mL^−1^	3.0 × 10^1^ ± 1.0 × 10^1^	< 100	< 100	< 1.0 × 10^1^	8.0 × 10^1^	7.0 × 10^2^	NE	NE	ND
Total heterotrophic bacteria THB	CFU mL^−1^	6.0 × 10^5^ ± 1.0 × 10^1^	4.5 × 10^4^ ± 3.0 × 10^1^	7.1 × 10^4^ ± 2.0 × 10^1^	2.3 × 10^6^	3.5 × 10^5^	7.8 × 10^6^			NE
Ammonium-oxidising bacteria AOB	CFU mL^−1^	< 100	< 100	2.2 × 10^1^ ± 1.0 × 10^1^	9.0 × 10^2^	9.0 × 10^2^	6.2 × 10^3^			

Sampling 1 (M-1): December 2019. Sampling 2 (M-2): July 2020. M-3: December 2020. M-4: April 2021. M-5: June 2021. M-6: December 2021. NE: Not specified. ND: Not to be detected

*Resolution 0631 Ministry of Environment and Sustainable Development 2015

**Resolution 1207 Ministry of Environment and Sustainable Development

***https://www.epa.gov/wqc/basic-information-water-quality-criteria and https://www.ecfr.gov/current/title-40/chapter-I/subchapter-N. 2023

BHT (7.8 × 10^6^ CFU mL^−1^), TC (4.1 × 10^4^ ± 1 × 10^1^ CFU mL^−1^), *E. coli* (7.0 × 10^2^ CFU mL^−1^) and AOB (6.2 × 10^3^ CFU mL^−1^) counts, occurred in this sampling (Table 5). Being of great interest the AOB since in none of the other sampling’s populations higher or equal to 1000 CFU mL^−1^ were recovered, an increase that was related to the NH_4_^+^ concentration detected in the WW (43 mg L^−1^), and these populations could enhance NH_4_^+^ oxidation by binding with *N. europea*, *N. oceani,* and *N. multiformis* co-inoculated strains.

### Assembly and evaluation of the biological reactor coupled to secondary sedimentation

WW sample from December 2021 served for the removal curves since the highest contamination levels it contained, especially for SS (4.0 mL L^−1^), COD (697 mg L^−1^), NH_4_^+^ (43 mg L^−1^), NO_2_^−^ (3.0 mg L^−1^) and NO_3_^−^ (8.5 mg L^−1^). Note that the concentrations of SS, COD and NO_3_, were above the maximum permissible levels for discharge and reuse of treated wastewater according to Colombian regulations; therefore, it was advisable to treat them before discharging them into the sewage system or reuse (Ministerio de Ambiente y Desarrollo Sostenible 2014, 2015). On the other hand, when comparing results from December 2021 sample with normative by the United States Environmental Protection Agency (EPA), the TSS, BOD_5_, and NH_4_^+^ concentrations did not comply with the Water Quality Standards (WQS) suggested by the EPA in the Code of Federal Regulations, Title 40, Chapter I, Subchapter N (https://www.epa.gov/wqc/basic-information-water-quality-criteria 2023; https://www.ecfr.gov/current/title-40/chapter-I/subchapter-N, 2023), (Table 5).

**Fig. 4:**
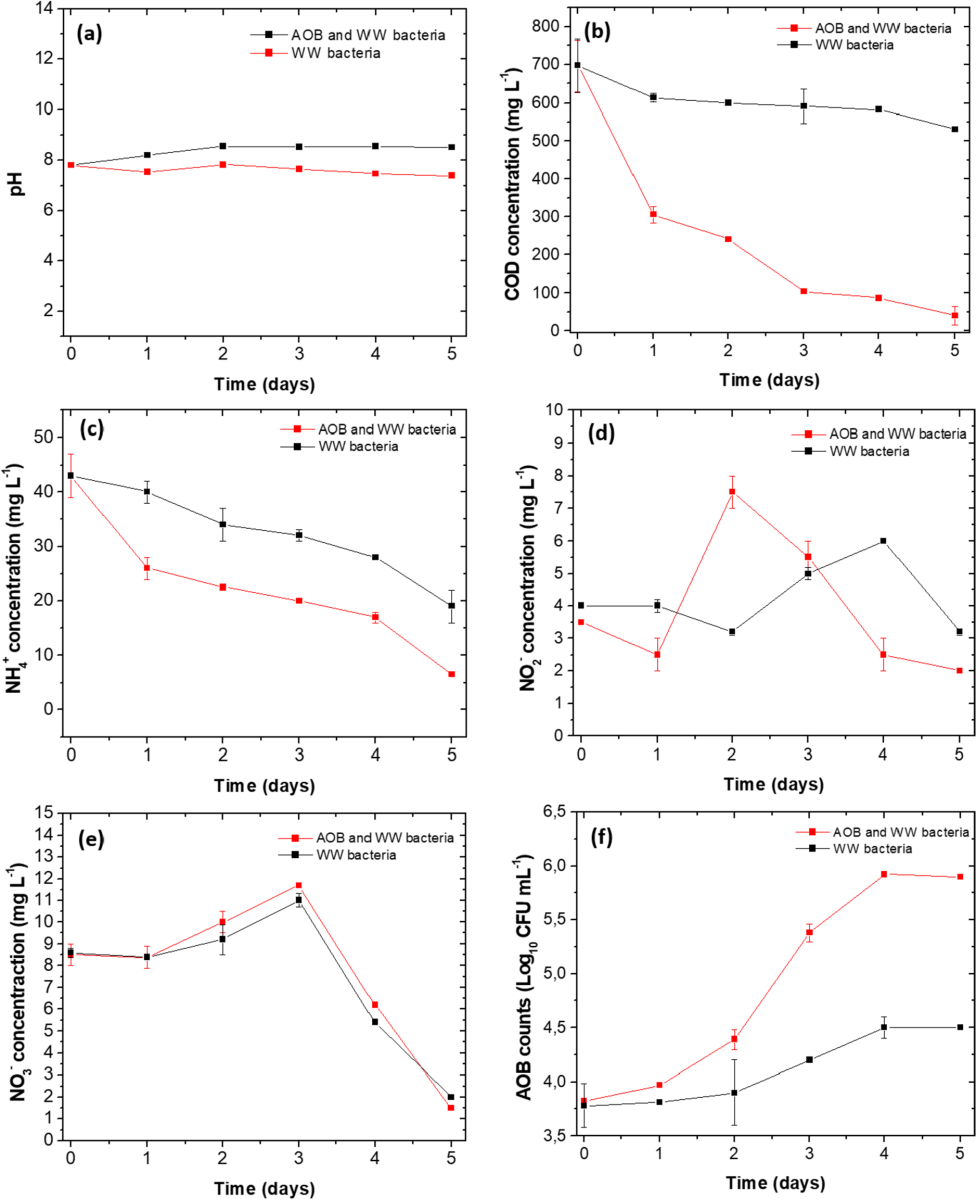
Removal curves in WW. **a** pH, **b** COD concentration, **c** NH_4_^+^ concentration, **d** NO_2_^−^ concentration, **e** NO_3_^−^ concentration, **f** AOB Counts. Average of three replicates with respective standard deviation

Concerning the pH behaviour as a function of time, the curves with AOB and WW bacteria showed an increase in pH (initial 7.0 ± 0.2), ending at 8.5 ± 0.2. In contrast, in the curve with only WW bacteria, the pH decreased slightly to end at 7.4 ± 0.2 (Fig. 4a). None of the pH values stayed outside the maximum permissible values for discharge according to Colombian regulations (6.0–9.0), (Ministerio de Ambiente y Desarrollo Sostenible 2015). The COD concentration decreased as a function of time, being more efficient when using AOB and WW bacteria, to end at 39.5 mg L^−1^, a value that is within the discharge standard (225 mg L^−1^), (Ministerio de Ambiente y Desarrollo Sostenible 2015). For the curve with WW bacteria, the final concentration was 531 mg L^−1^, a value approximately 13.5 times higher than the obtained one in the removal curve with AOB addition (Fig. 4b).

The behaviour of the inorganic nitrogen substrate (initial NH_4_^+^ concentration: 43 mg L^−1^) showed a decrease in both curves. However, when using the consortium of the three AOB for the WW bacteria, a remarket decrease in NH_4_^+^ concentration occurred, ending with 6.5 mg L^−1^ after five days of treatment. In contrast, in the curve with only WW bacteria, the final NH_4_^+^ concentration was 19 mg L^−1^, a value approximately three times higher than in the removal curve with the addition of the AOB consortium (Fig. 4c).

The oxidation of NH_4_^+^ generated the production of NO_2_^−^ in the first stage; for this reason, an increase in the concentration of NO_2_^−^ happened during the first two days (curve in which AOB consortium) (Fig. 4d). Subsequently, its concentration decreased to end at (2.0 mg L^−1^). In the curves with only WW bacteria, an increase in NO_2_^−^ and a subsequent decrease occurred, but between days 4 and 5 (final concentration 3.2 mg L^−1^), suggesting that AOB bacteria were present within the WW bacteria and in agreement with the microbiological analyses performed on the WW before starting the removal experiments (Fig. 4d, Table 5).

In relation to the production of NO_3_^−^ as the final product of NH_4_^+^ oxidation, the initial concentration was 8.5 mg L^−1^ during the first 24 h no changes appeared in any removal curves. Between days 2 and 3, the NO_3_^−^ concentration increased similarly in both curves, and by days 4 and 5, a decrease was observed to end at 1.5 and 2.0 mg L^−1^ for AOB and WW and only WW bacteria curves (Fig. 4e). The concentrations were below the values required by Colombian regulations (5.0 mg L^−1^), (Ministerio de Ambiente y Desarrollo Sostenible 2015).

In both removal curves, the initial AOB counts were 3.82 and 3.78 Log_10_ de CFU mL^−1^; during the first day, the AOB grew slightly, and no marked differences appeared (Fig. 4f). After two days in the curve with AOB and WW bacteria, a higher growth was observed, ending at 5.92 Log_10_ CFU mL^−1^ after five days. In the curve, involving only WW bacteria, growth was slower and lower, ending at concentrations of 4.6 Log_10_ CFU mL^−1^ at five days (Fig. 4f). Additionally, initial and five-day counts were made for total heterotrophic bacteria (THB), obtaining initial counts of 7.1 and 6.0 Log_10_ CFU mL^−1^, for AOB and WW bacteria and only WW bacteria, respectively. After five days, the counts were 9.3 and 8.1 Log_10_ CFU mL^−1^ for AOB and WW bacteria and only WW bacteria. The increases were related to the use of organic matter from the WW, expressed as COD (697 mg L^−1^) and BOD_5_ (325 mg L^−1^). The final treatability ratio (BOD_5_/COD) was 0.46, so close to 0.5, indicating that the WW contained organic components that could be utilised by heterotrophic bacteria as carbon and energy sources and that WW had a medium biodegradability (Table 5).

## Discussion

### Preservation of bacteria and growth conditions

Ammonium oxidising bacteria (AOB) are a group of microorganisms involved in the oxidative stage of the biogeochemical nitrogen cycle and have valuable (van Kessel et al. 2015) environmental applications in soil and wastewater treatment (Nsenga Kumwimba and Meng 2019; Wright and Lehtovirta-Morley 2023; Zhang et al. 2024). These bacteria are involved in the transformation of NH_4_^+^ produced from nitrogenous organic matter, urea, inorganic fertilisers and quaternary ammonium-based disinfectants (Dong et al. 2017; Fatimazahra et al. 2023; Wright and Lehtovirta-Morley 2023). AOB are chemoautotrophic bacteria with slow growth rates, form small colonies, and are sensitive to physical and chemical changes both in their natural ecosystem and “in vitro” culture (van Kessel et al. 2015; Soliman and Eldyasti 2018; Elmoutez et al. 2023). As the genera of bacteria used in this paper are difficult to cultivate and maintain "in vitro", a preservation protocol had to be implemented. The most reported preservation involved the microcolony subcultures, the calcium alginate immobilisation, or cryopreservation in liquid nitrogen. Cryopreservation could use cryoprotectant substances such as dimethylsulfoxide (DMSO), dimethylsulfoxide with trehalose (DMSO/TT), and polyvinyl alcohol (Hoefman et al. 2013; Fujitani et al. 2015; Dong et al. 2017). These protocols are successful in maintaining the viability and activity of AOBs. However, cryopreservation employing 30% (w/v) glycerol and freezing at − 20 °C has not been widely studied for this type of bacteria, being an alternative for laboratories, which do not have freezing systems at − 80 °C or ultra-freezing with liquid nitrogen. The preservation at − 20 °C showed at five months that it is a promissory alternative for preserving *N. europea*, *N. oceani* and *N. multiformis* (Fig. 1a, b and c). Glycerol acts as an intracellular and extracellular cryoprotectant in two known ways; the external coating of the cells and the internal passing through the cell membrane (penetrating protectant) to decrease the formation of large, sharp intracellular water crystals (Meza et al. 2004; Siddiqui et al. 2015). Additionally, glycerol's advantage is that it reacts by forming hydrogen bonds with the intracellular water to protect the cells (Poutou et al. 1994; Mahmmoud 2020). For these reasons, counts did not decrease by more than two log units at baseline, 24 h and five months of preservation (Fig. 1a, b and c).

Likewise, the preservation method does not affect the biological activity of the three bacteria used; observing that before and after cryopreservation, *N. europea*, *N oceani* and *N. multiformis* performed the oxidation of the inorganic substrate (NH_4_^+^) and produced NO_2_^−^, by the action of Ammonia Monooxygenase (E.C. 1.14.99.39) and Hydroxylamine Oxidoreductase (E.C. 1.7.2.6), (Zhu et al. 2022). These intracellular enzymes are in the cell membrane; the glycerol preservation method protects the cell membrane from the ice crystal formation, which forms rapidly and tends to be blunt when flash-frozen at – 20 °C (Soliman and Eldyasti 2018; Zhu et al. 2022).

The results obtained before and after implementing a preservation method are similar to those obtained by Hoefman et al., (2013); in their research, authors preserved different ammonium-oxidising bacteria using dimethyl-sulfoxide (DMSO), DMSO with trehalose and freezing at – 80 °C to compare with preservation in liquid nitrogen. The authors observed that DMSO with trehalose preserved and maintained the activity of the bacteria better than liquid nitrogen, making it a less expensive and more accessible alternative in laboratories (Hoefman et al. 2013). When comparing Hoefman's results with the present study, it stands out that in the present study, a less expensive cryoprotectant and easy to acquire, such as glycerol, allowed preservation. On the other hand, the lower temperature (− 20 °C) than that evaluated by Hoefman (− 80 °C) represents an operational advantage since a − 80 °C ultracold freezer is not available in all research and service laboratories.

### Plackett–Burman experimental design (PBED)

For "in vitro" AOB cultivation, an oligotrophic medium containing at least one ammonium salt, phosphates, and trace elements and the presence or absence of an inorganic carbon source should be used (Soliman and Eldyasti 2018). Regarding operating conditions, AOBs need aeration/agitation, pH close to neutral and temperatures that do not exceed the optimal value for growth and enzymatic activity (Hoefman et al. 2013; Fujitani et al. 2015; Nsenga Kumwimba and Meng 2019); conditions simulated at T5 (6607 mg L^−1^ of (NH_4_)_2_SO_4_, 84 mg L^−1^ CaCO_3_, 40 mg L^−1^ MgSO_4_·7H_2_O, 40 mg L^−1^ CaCl_2_·2H_2_O and 200 mg L^−1^ KH_2_PO_4_, 10% (w/v) inoculum, no copper addition, pH 7.0 ± 0.2, 200 r.p.m., 30 days) of the PBED (Table 1), which resulted in high NO_2_^−^ concentrations (0.323 ± 0.003 mg L^−1^) and some of the highest colony count (6.2 Log_10_ CFU mL^−1^), (Fig. 2). The increases in the concentration of NO_2_^−^ mg L^−1^ and CFU mL^−1^ obtained in treatment five were related to two aspects: First, the AOB uses ammonium as an energy source through its oxidation, which corresponds to the first stage of nitrification (Aizawa et al. 2023). In turn, these bacteria obtain carbon from inorganic sources such as CO_2_ or mineral substitutes such as carbonates, obtaining the carbon necessary for proteins, lipids and amino acids to generate new cells (Gonzalez-Cabaleiro et al. 2019).

Increasing ammonium sulphate concentration from 500 mg L^−1^ (basal medium) to 6607 mg L^−1^ (PBED T5 medium) favoured growth and NO_2_^−^ production because more NH_4_^+^ (1799 mg L^−1^ NH_4_-N) was supplied for energy and as a source of nitrogen for the production of new cell compounds (Soliman and Eldyasti 2018). Authors such as Van Kessel et al., (2015) observed that increasing NH_4_^+^ from 100 to 600 µM increased NO_2_^−^ production, and these increases also regulated the AOB participation and nitrite-oxidising bacteria (NOB) populations (van Kessel et al. 2015). At pH 7.0 ± 0.2, the NH_4_^+^ is available in its ionic form, favouring the interaction of the substrate and the catalytic centre of Ammonia Monooxygenase (E.C. 1.14.99.39), (Stein et al. 1997; Bao et al. 2017; Huang et al. 2017; Antileo et al. 2022); at this pH close to the optimal one for AOB, higher amounts of cells recovered from non-saline surface waters could be produced (French et al. 2012; Másmela-Mendoza et al. 2019).

Although under the experimental conditions evaluated in the PBED, factors E (percentage of inoculum), F (CaCO_3_) and G (agitation) did not have a significant effect on the response variables (*p* > 0.0001), they are crucial for pure cultures growth of AOB under laboratory conditions. AOB such as *N. europea* are β obligate chemolithotrophic proteobacteria, which obtain energy from the oxidation of ammonium, using O_2_ as a final electron acceptor; therefore, proper agitation favours the transfer of O_2_ from the aqueous phase into contact with the cells (Peng et al. 2015; Zorz et al. 2018; Gonzalez-Cabaleiro et al. 2019). On the other hand, carbon for anabolism of new cellular compounds is obtained by CO_2_ fixation using the Calvin–Benson–Bassham (CBB) cycle (Zorz et al. 2018; Gonzalez-Cabaleiro et al. 2019). However, the assimilation of this carbon form is slow and limited by low availability in an aqueous medium (Hoefman et al. 2013; Gonzalez-Cabaleiro et al. 2019). To solve this carbon accessibility, calcium or sodium carbonate can be added to culture media as an inorganic carbon source to complement CO_2_. By having two carbon sources, more initial cells (percentage of inoculum) and agitation (a form of aeration), AOB grows in greater quantity, which will become reflected in an increasing amount of NH_4_^+^ oxidised (Peng et al. 2015; Dong et al. 2017; Gonzalez-Cabaleiro et al. 2019).

Although the results obtained in the Plackett Burman design were promising and allowed for improvement in the culture medium previously reported by Martínez et al. (2013), the limitation of the PBED involves the number of levels evaluated, as it allows only one high-level (+ 1) and one low-level (− 1), while the design inclusion of 3–5 central points can amplify the design. Whether necessary to get a precise effect value of large factors number, the results of a Plackett Burman should serve for feeding an experimental optimisation design as a complement, such as a response surface model, such as Box–Behnken Experimental Design (BBED). On the other hand, was not studied the metal ions effect (Cu^2+^, Zn^2+^ and Mn^2+^). Metal ions can be positive for the relative abundance of AOB in aqueous media (Fan et al. 2022). Even so, PBED results allow the establishment of successful modification for a culture medium because 15 days of culture reached high biomass concentrations of a demanding bacterium such *as Nitrosomonas europea*.

### Growth curves in modified ammonium broth using *Nitrosomonas europea*, *Nitrospira multiformis* and *Nitrosococcus oceani*

One of the critical points limiting the use and commercialisation of pure strains of AOB for wastewater treatment is that their propagation in oligotrophic media is slow, and long production times are required (Dionisi et al. 2002; Song et al. 2021), which can be solved when culture medium and operating conditions are improved and accompanied by strict bioprocess control. Dionisi et al., (2002), Song et al., (2021) and Li et al., (2023) reported that these bacteria are slow-growing and very sensitive to environmental factors such as pH, O_2_ concentrations, presence of toxic substances and initial ammonium concentration; which determines that sludge in treatment plants requires a longer time than heterotrophic microorganisms to reach a stable nitrification. For these reasons, an alternative is the external production of these bacteria under controlled conditions, which are then introduced into the biological reactors, part of the secondary wastewater treatment.

In the growth curves, *N. europea*, *N. oceani* and *N. multiformis* were evaluated in an oligotrophic medium (6607 mg L^−1^ of (NH_4_)_2_SO_4_, 84 mg L^−1^ CaCO_3_, 40 mg L^−1^ MgSO_4_·7H_2_O, 40 mg L^−1^ CaCl_2_·2H_2_O and 200 mg L^−1^ KH_2_PO_4_) for 30 days), improved by the supplementation with higher concentrations of ammonium sulphate (6607 mg L^−1^) and calcium carbonate (84 mg L^−1^). These concentrations served as a source of energy and carbon to support bacterial growth. According to results presented in Fig. 3a, each of them performed differently, with *N. europea* being the most efficient (Fig. 3c, d, Table 4). The reasons why *N. europea* was superior in several respects comes of the wide distribution in aquatic and terrestrial environments and the adaptation capacity to different environmental conditions, including tolerance to high NH_4_^+^ concentrations such as those evaluated in this article (Hughes et al. 2021). *N. europea* employs simple diffusion systems to internalise NH_4_^+^, a transport system that has lower energy expenditure for the cell and allows *N. europea* to obtain energy more rapidly from the oxidation of ammonium sulphate (Keerio et al. 2020). On the other hand, *N. europea* has a higher affinity for NH_4_^+^ and can reach *K*_*M*_ values ranging from 12.5 and 160 μM of NH_4_^+^ (Sedlacek et al. 2019). Finally, *N. europea* produces more varieties of terminal oxidases (such as cytochrome c, cytochrome aa3, and low-affinity terminal oxidase, among others) than the other AOB studied, which serve it to better oxygen concentration changes adaptation in its environment (Sedlacek et al. 2019). These enzymes are crucial since the dissolved oxygen concentration in the culture medium must be above 2.5 mg L^−1^ to avoid cell growth and NH_4_^+^ oxidation inhibition (Pellitteri-Hahn et al. 2011; Peng et al. 2015). Hughes et al., (2021) had similar results to ours when employing *N. europea* and *N. oceani* as AOB involved in the production of IO^3−^ in the presence of I^−^ as the sole source of Iodine; they cultured the two bacteria in seawater, supplemented with ammonium chloride and observed that *N. europea* produced more cells mL^−1^ at eight days than *N. oceani* at 12 days of culture (Hughes et al. 2021).

Concerning *N. oceani*, satisfactory growth and NH_4_^+^ oxidation showed at 18 days (Fig. 3a, Table 4). However, it was lower than for *N. europea*. This bacterium is of marine origin in this ecosystem oligotrophic conditions predominate, with low NH_4_^+^ concentrations, slightly alkaline pH and higher concentration of solutes (Zorz et al. 2018; Hughes et al. 2021). Unfortunately, these results were quite different at present work when strain growth occurred in the modified ammonium sulphate broth. According to studies by Campbell et al. (2011) and Hughes et al. (2021), *N. oceani* grows best in NH_4_^+^ concentrations ranging from 8 to 12.5 mM, ammonium sulphate and ammonium chloride, concentrations 4 and 6.3 times lower than those used in this study (Fig. 3c, d, Table 4), (Campbell et al. 2011; Hughes et al. 2021). On the other hand, the initial pH of the culture medium setup was 7.0 ± 0.2, a value slightly lower than the optimal value reported for *N. oceani* (7.6–8.0 ± 0.2), which could affect the enzymatic activities related to ammonium oxidation (Fig. 3b), (Hughes et al. 2021).

The lowest results associated with colony counts, NO_2_^−^ production, yields and productivities were obtained with *N. multiformis*, determining that its exponential phase took 21 days (Fig. 3a, Table 4). *N. multiformis* is a neutrophilic AOB with an optimal growth temperature ranging from 25 to 30 °C, pH 7.5 ± 0.2 and ammonium concentrations between 5 and 10 mM (Huang et al. 2018). However, other authors have isolated *N. multiformis* from several environments, such as acidic pH and low-temperature (10–20 °C) soils (Huang et al. 2018; Sanders et al. 2019) In the present study, a possible reason for its reduced growth was the high ammonium concentration that led to decreased growth and ammonium oxidation (Fig. 3a, b, d, Table 4).

#### NH_4_^+^ removal curves in wastewater (WW)

##### WW sampling and characterisation

At the end of the months of total confinement due to the SARS-CoV-2 pandemic, the return to the presence and the rigorous maintenance of biosecurity measures for the population generated increases in the amount of domestic wastewater, changes in its composition and release of byproducts derived from chlorine, quaternary ammonium, among others (Hora et al. 2020; Wang et al. 2021). In the present study, this increase was evident when analysing the six sampling periods (end of 2019 to end of 2021) and the highest values were detected at the end of the second half of 2021 (Table 5).

Several authors have reported physical and chemical wastewater parameters changes during the SARS-CoV-2 pandemic. Ahmed et al., (2022), characterised different WW during the years 2015–2020, observing variations in COD concentration, ammonia nitrogen, available phosphorus, pH and solids, attributed to the mixing of domestic water with hospital wastewater that had high concentrations of pharmaceutical compounds, disinfectants, surfactants and detergents containing phosphorus (Ahmed et al. 2022). Nasseri et al., (2021) studied the existence of SARS-CoV-2 in untreated and post-treated domestic wastewater (DWW) in three cities in Iran. Authors detected the virus in the influents of treatment plants, a marked variation in COD, BOD_5_ and SST, which was related to the number of people generating wastewater in each city, higher consumption of drinking water and higher use of personal care products (Nasseri et al. 2021).

In the biological reactors with activated sludge used for the aerobic removal of NH_4_^+^ in WW, occurred autotrophic nitrification (Song et al. 2021), Comamox (Gonzalez-Martinez et al. 2016; He et al. 2022) and aerobic or facultative denitrification (Duan et al. 2015; Song et al. 2021) processes. In this kind of reactor, the number and biological activity of AOB and NOB bacteria are considered as one of the limiting steps in the conversion of ammonia nitrogen because they determine the minimum maturation or stabilisation time of the sludge to initiate the nitrification process (Dionisi et al. 2002; Song et al. 2021).

The present research shows that co-inoculation of *N. europea*, *N. oceani* and *N. multiformis*, in a possible cooperation with WW heterotrophic bacteria and AOB, improved the removal of COD and NH_4_^+^ (Fig. 4b, c). With more AOB, NH_4_^+^ has oxidised more efficiently; consequently, the concentration of NO^2−^ increased after the first 24 h (Fig. 4d). This intermediate started to decrease from the second day onwards, suggesting the presence of NO_2_^−^ oxidising bacteria (NOB) that could be present in the WW (not quantified in this study). On the same day, an increase in NO_3_^−^ concentration was observed, which helps to support the possible presence of NOB bacteria in the WW (Fig. 4e). Gonzales-Martinez et al. (2016) reported that microorganisms such as *Nitrobacter* sp., *Nitrospira* sp., *Nitrococcus* sp., and *Nitrospina* sp., can be recovered from WW. However, *Nitrospira* sp., the predominant NOB, participates in the second stage of autotrophic nitrification and has been related to Comamox processes (Gonzalez-Martinez et al. 2016; He et al. 2022).

On the other hand, from day three onwards, a decrease in NO_3_^−^ concentration occurred; this intermediate can be assimilatively reduced by microorganisms for new cellular compounds production or reduced to N_2_ under anoxic conditions (Fig. 4e). This transformation is not frequent in an aerobic activated sludge reactor where O_2_ concentration, should be maintained between 2.0 and 3.0 mg L^−1^ (Pellitteri-Hahn et al. 2011; Peng et al. 2015). However, within the organic and inorganic aggregates of the sludge, certain oxygen-free zones are formed, and complete denitrification can occur (Ren et al. 2020b; Moloantoa et al. 2022). Another combined mechanism by which nitrification and denitrification remove NO_3_^−^ in an activated sludge reactor is due to the participation of microorganisms that carry out both processes under aerobic conditions. These microorganisms can be recovered from wastewater and include genera such as *Acinetobacter* sp., *Bacillus* sp., *Klebsiella* sp., *Escherichia* sp., *Enterobacter* sp., *Pseudomonas* sp., among others (Song et al. 2021). Although the removal experiments only quantified the total heterotrophic bacteria at the beginning and after five days, it shows an increase, with values of 9.3 and 8.1 Log_10_ CFU mL^−1^ for the curves with AOB and WW bacteria and only with WW bacteria. In the initial characterisation of WW, were also detected HTB, TC and *Escherichia coli* (Table 5). Bacteria that are part of the TC, especially *E. coli*, could also participate in the NO_3_^−^ removal.

## Conclusions

In this study, a modified oligotrophic medium and operational conditions was obtained that attained an 8.23 ± 1.9, 7.56 ± 0.7 and 4.2 ± 0.4 Log_10_ CFU mL^−1^ of *Nitrosomonas europea*, *Nitrosococcus oceani*, and *Nitrosospira multiformis*. Furthermore, the results demonstrated that bio-augment of the biomass of the studied bacteria in consortium with heterotrophic WW bacteria allow the aerobic removal of COD (final concentration of 39.5 mg L^−1^) and nitrogen cycle intermediates (NH_4_^+^, NO_2_^−^, and NO_3_^−^ final concentration of 6.5, 2.0 and 1.5 mg L^−1^) present in the studied wastewater. These results are a promising alternative for others type of wastewater such as, hospital, pig farms and poultry farms.

### Supplementary Information

Below is the link to the electronic supplementary material.

Supplementary file1 (DOCX 2300 KB)
