# Supplementary material for: Wastewater treatment from a science faculty during the COVID-19 pandemic by using ammonium-oxidising and heterotrophic bacteria
Source: 3 Biotech. 2024 Apr 9;14(5):129. doi: 10.1007/s13205-024-03961-4 (PMC11003938; doi:10.1007/s13205-024-03961-4)
Supplement: Supplementary file 1 — Supplementary file1 (DOCX 2300 KB) [file 13205_2024_3961_MOESM1_ESM.docx]

**Supplementary Material**

**Fig. S1 Macroscopic and microscopic characteristics of *Nitrosomonas europea***

*Nitrosomonas europea* grew on ammonium agar for ten days at 30 ^o^C. (Fig. S1a) Macroscopic morphology, small, punctate colonies with a slight bluish tinge in the light. (Fig. S1b) Microscopic morphology Gram-negative coccobacilli.


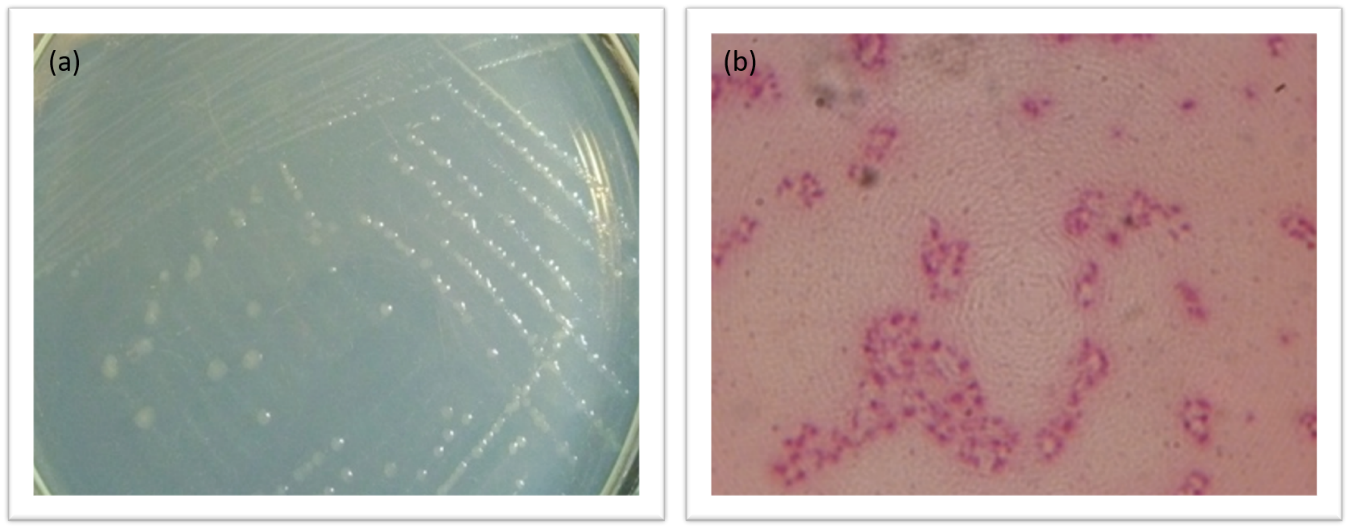


**Fig. S2 Macroscopic and microscopic characteristics of *Nitrosococcus oceani*** *Nitrosococcus oceani* grew on ammonium agar for ten days at 30 ^o^C. (Fig. S2a) Macroscopic morphology, small, punctiform colonies with a slight bluish tinge in the light. (Fig. S2b) Microscopic morphology Gram-negative coccobacilli.


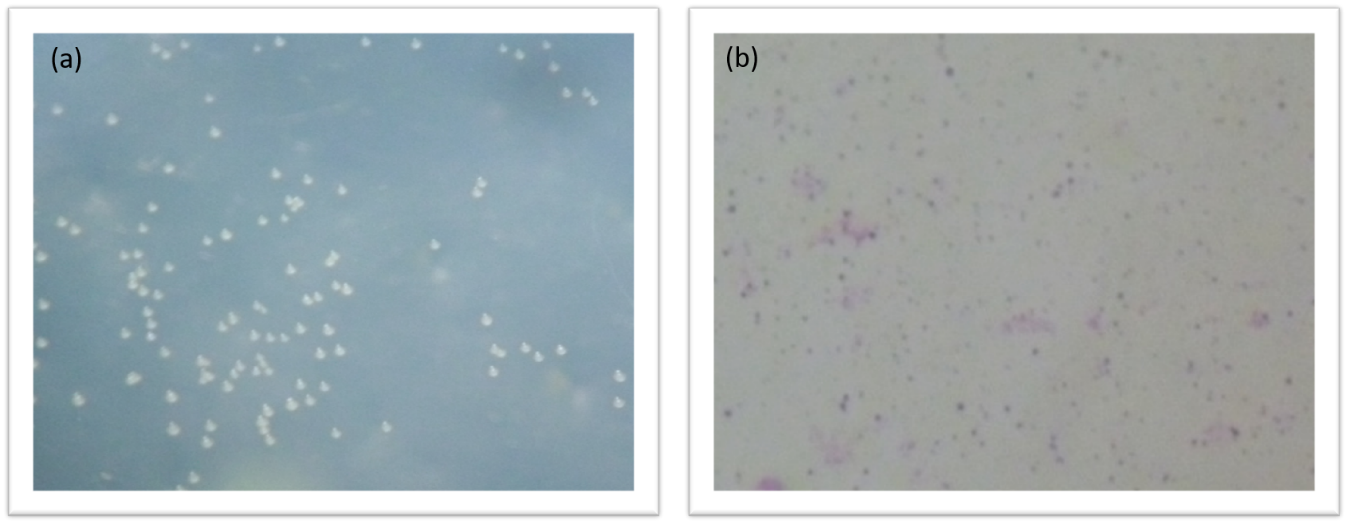


**Fig. S3 Macroscopic and microscopic characteristics of *Nitrospira multiformis***

*Nitrospira multiformis* grew on ammonium agar for ten days at 30 ^o^C. (Fig. S3a) Macroscopic morphology, colonies are small, punctate and in light, they appear slightly bluish. (Fig. S3b) Microscopic morphology Gram-negative coccobacilli.


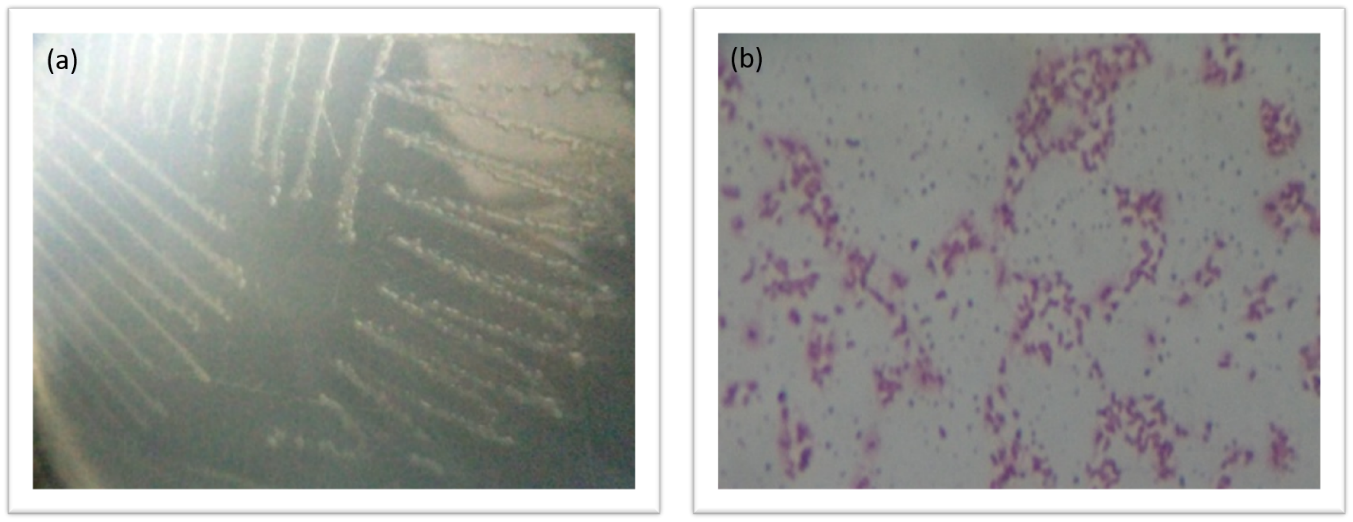


.
